# Retreatment or replacement of previous endodontically treated premolars with recurrent apical periodontitis? An 8-year historical cohort study

**DOI:** 10.1007/s00784-025-06238-z

**Published:** 2025-03-13

**Authors:** Fausto Zamparini, Andrea Spinelli, Jacopo Lenzi, Ove A. Peters, Maria Giovanna Gandolfi, Carlo Prati

**Affiliations:** 1 Endodontic Clinical Section, Dental School, Department of Biomedical and Neuromotor Sciences, University of Bologna, Bologna, Italy; 2 Laboratory of Green Biomaterials and Oral Pathology, Dental School, Department of Biomedical and Neuromotor Sciences, University of Bologna, Bologna, Italy; 3 Department of Biomedical and Neuromotor Sciences, University of Bologna, Bologna, Italy; 4 School of Dentistry, The University of Queensland, Herston, QLD Australia

**Keywords:** Decision making, Historical cohort study, Root canal retreatment, Implant placement, PAI, MBL

## Abstract

**Objectives:**

The study evaluated previously-endodontically-treated premolars affected by periapical lesions and/or secondary caries requiring a multidisciplinary decision between (non-surgical) retreatment or extraction and implant replacement over an 8-year minimum follow-up.

**Materials and methods:**

The decision-making was performed among a pool of patients attending a University Dental School. All patients presented at least one failing previously endodontically treated premolar. Recorded parameters were: structural conditions (residual coronal-structure, caries), periodontal and endodontic status (CEJ-MBL, initial-PAI, post-presence). Two experienced operators made the decision-making and classified teeth as retreatable and restorable (Endo-group) or suitable for extraction and implant replacement (Implant-group). Logistic regression and Cox-proportional-hazard analyses with clustered-standard-errors compared baseline-characteristics and treatment-outcomes. Odds-ratios (ORs) with 95% confidence-intervals (CIs) were reported for baseline-characteristics. Hazard-ratios (HRs) expressed the association of treatment-groups with time-to-event.

**Results:**

Ninety-six patients (*n* = 124 premolars) were enrolled (49 M;47 F; mean-age 53.1 ± 11.6 years). The decision-making splitted 54.8% treatments to Endo-group (*n* = 68) and 45.2% to Implant-group (*n* = 56). The 8-year survival were 85.1% for Endo-group and 98.2% for Implant-group. The 8-year success were 80.5% and 93.9%. The HR from Cox regression favored Implant-group (HR = 0.12, *P* = 0.049). The Endo-group showed the highest number of critical complications (15%) due to fractures, despite the healing of lesions. Implant-group had a higher percentage of minor prosthetic complications (14%).

**Conclusions:**

Endo-group demonstrated higher percentage of critical complications compared to Implant-group during the follow-up. Root fractures were accounted as main responsible, while periapical disease did not affect healing, survival and clinical longevity.

**Clinical significance:**

Insufficient crown structure was the major parameter associated with root fracture. In these cases, implant replacement strategy represented an adequate therapy justified by the higher success compared to root canal retreatment.

## Introduction

One of the clinical problems that frequently afflicts the daily clinical routine is the evaluation of clinical treatment and the fate of seriously compromised teeth affected by periapical lesion, alteration of crown structure and restoration defects [1]. The presence of a previous root canal treatment and exacerbated periapical lesions increase the number of procedures and, as consequence, the need for a critical correct choice [1, 2]. As results, root canal retreatment and extraction and implant placement could be both considered and recommended, requiring the operator to select the best option and to predict the longevity of the treatment, eventual complications, risks and factors as pain, cost and number of appointments to complete the therapy [3]. Nevertheless, which treatment is more effective remains an open question.

The success rates for retreatments ranged from 65 to 85% over a ten years observation period [4–13] as shown in recent systematic review and meta-analysis on endodontic retreatments. Advances in both instrumentation and filling techniques did not significantly influence these values [1, 14]. On the other hand, implant rehabilitations demonstrate high survival and success rates over 6–10 years, ranging from 90 to 96% according to previously available clinical studies [15–19]. The clinical decision-making is therefore critical and requires many considerations to analyse the numerous factors affecting the longevity of teeth and treatment efficacy [20–24].

Non-surgical retreatments complications include persistent periapical exacerbations and apical reinfections during follow-up, often attributable to the recontamination of the endodontic space or to the incomplete removal of infected tissues [1, 2]. When not possible, surgical interventions (i.e. apical surgery) is necessary to remove the persistent apical lesion and to prolong the life of the endodontically-treated tooth [25].

Conversely, implant complications are lower in number, but could rapidly influence the long-term prognosis. Early implant biological complication (such as persistent inflammation of the peri-implant soft tissues, local infections and chronic occlusal overload) critically affects osseointegration phases and the rehabilitation longevity [25, 26].

The longevity of both teeth and implant rehabilitation are influenced by patient specific factors, including systemic health conditions, oral health status, supportive periodontal therapies and occlusal patterns, which could adversely affect the success of the treatments [27]. Different research groups [3, 5, 28, 29] suggested that experience, dental specialties, clinical background and treatment philosophies of the operator play a critical role and directly influence the clinical treatment [3, 30–32]. Teeth morphology, root anatomy, age, and systemic conditions of the patients may also influence the decision-making [1, 6].

Previous Authors proposed a helpful index to assist operators in the decision-making [33], but the complexity of endodontic and non-endodontic clinical parameters - such as lack of coronal integrity, presence of crowns, intraradicular posts, periodontal and periapical infections - still represent masking factors and confounding conditions that increase the risk for errors [33]. It is difficult to directly compare the outcome of retreatments versus dental implant rehabilitations and, limited data from a low number of studies are available [29, 34, 35]. The different method to analyse and to compare the two options produced several discrepancies and incomplete results [29, 34, 35].

This historical cohort study evaluated previously endodontically-treated premolars affected by periapical lesion and/or deep secondary caries which required a multidisciplinary critical evaluation based on a (non-surgical) retreatment or extraction and implant rehabilitation. Their survival and success rates, complications and clinical parameters were compared in a clinical 8-year follow-up. The authors conceived a Decision-Making score (DM-Score) to determine the most suitable treatment on the basis of different structural, periodontal, and endodontic parameters.

## Materials and methods

### Study setting and patient selection criteria

The study was planned as a non-randomized historical prospective study on a cohort of patients followed for 8 years. The study was approved by the institutional ethical committee (CE AVEC ENDO IMPLANT RETRO 10.22) and registered in Clinical Trials.gov (NCT06250114). This work was written according to the STROBE guidelines for observational epidemiological studies [36] (Figure S1) and was conducted in full accordance with ethical principles, including the Declaration of Helsinki [37]. An informed consent was obtained from each participant.

Patients treated in the period from January 2007 to December 2015 were eligible. Demographic information including age, gender, smoking, and general health conditions were recorded. Patients were assigned to the clinical protocols and recall programme if they fulfilled the following inclusion criteria:

- Good general health (ASA status 1–2);
- Age between 18 and 75 years;
- Local geographic provenience;
- Similar socio-economic condition (medium-high education and economic level) [38];
- Presence of at least one premolar with a failing root canal treatment with an uncertain decision making (to perform a nonsurgical retreatment or extraction and implant rehabilitation);
- Possibility to attend to regular annual maintenance visits.

Exclusion criteria were the following:

- General contraindication to implant surgeries;
- Absence of a previous root canal treatment on the affected tooth;
- Teeth that would serve as unit of multiple prosthetic rehabilitation;
- Presence of vertical root fractures that lead to the impossibility to perform a secondary root canal treatment.
- ASA score > 2;
- Diabetes or any condition that could compromise bone healing or immune response;
- Pregnancy, or breast feeding;
- Heavy smoking (> 10 cigarettes/day);
- Exposure to radiation therapy focused on the head and neck region;
- Malignant disease directly involving the jaws.

**Fig. 1:**
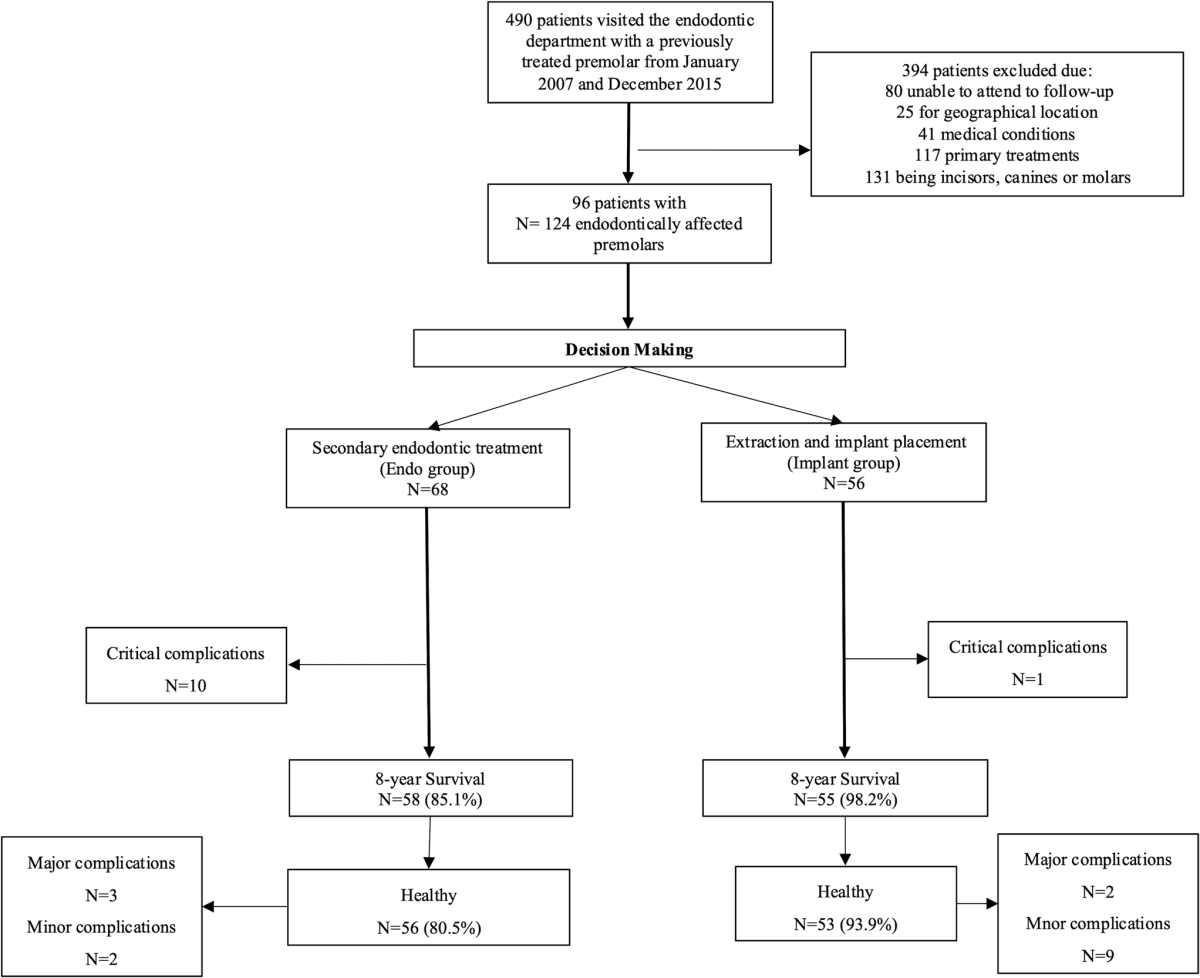
Flow chart depicting patient decision making and final 8-year outcome

The final evaluation was performed between January 2019 and December 2023 (Fig. 1).

### Retreatment versus extraction and implant replacement

The primary consideration for inclusion in the study was that both approaches had a sound clinical and biological rationale and proved acceptable in terms of cost for the patient.

Nevertheless, the final decision was always made by two operators based on their experience and adherence to evidence-based dentistry and best clinical practices [39].

The following parameters were considered and recorded for the decision-making process of each treatment:

- Structural parameters: residual coronal structure (3 or 4 walls, 1 or 2 walls, 0 walls, full prosthetic reconstruction); presence of deep carious lesions (yes, no).
- Periodontal parameters: distance between cervical enamel junction and first bone contact (CEJ–MBL) (≤ 3 mm, > 3 to < 6 mm, ≥ 6 mm) [24].
- Endodontic parameters: initial PAI (1 or 2, 3 to 5); presence of post-retained core (yes, no).

### Retreatment and post-endodontic reconstruction phases (Endo group)

Non-surgical root canal secondary treatment was performed in multiple visits and following standardized clinical protocols [9, 40]. All procedures were performed by clinical tutors of the endodontic master programme in multiple appointment. Reasons for retreatment included cases of re-exacerbated periapical lesions due to an incomplete filling of the root canal, radiographical evidences of missed canals or infiltration of the coronal restoration. When causes of apical re-exacerbation were not clear, a cone beam CT (CBCT) was performed prior to treatment.

In all cases, local anaesthesia was obtained, dental dam isolation was achieved, and a straight-line access was prepared using diamond burs mounted on high-speed water-cooled handpieces (W&H, Bürmoos Austria). Ultrasonic tips (StartX, Dentsply Maillefer) were used to remove any existing metal and fiber posts under magnification. Gutta-percha solvents (Endosolv E or Endosolv R, Septodont, Saint-Maur-des-Fossés, France) were used to soften the obturation core and an initial entry into the canal was made with Gates-Glidden burs #3 – #4 (Dentsply Maillefer, Ballaigues, Swiss) to approximately 5–6 mm depth in the gutta-percha. A K-file crown-down instrumentation technique was employed. Working length was established with the aid of an electronic apex locator (Root ZX, Morita, Tokyo, Japan) and radiographically confirmed. Between appointments, teeth were temporarily restored with a non-eugenol provisional dressing (Coltosol, Coltene AG, Altstätten, Switzerland) associated to a cotton pellet without the placement of intracanal medicament between [9, 40].

The irrigation protocol included a total amount of 5.0 mL of 5% NaOCl. In case of root calcification, each canal was subjected to further irrigation with 3 min of 1.0 mL 17% EDTA solution. Final flush was performed using 2.0mL sterile water solution. A carrier-based obturation technique (Thermafil, Dentsply DeTrey, Konstanz, Germany) with epoxy resin-based sealer (AH Plus, Dentsply Dental Trey) was used to complete the root canal filling. A temporary obturation was performed allowing the root canal sealer to completely set before final obturation procedures. Two to 3 weeks following root canal filling, a self-etching adhesive system (SE Bond, Kuraray Co., Ltd, Osaka, Japan) was applied. A light-curable flow (Gradia Direct Flow, and G Aenial Flo GC, Leuven, Belgium) was applied with bilaminar technique to fill the cavity floor / pulp chamber and a composite resin (Gradia Posterior and G Aenial Posterior, GC, Leuven, Belgium) was applied in a multilayering technique to complete the build-up.

Provisional crowns were prepared after 3 months from tooth build-up and fixed with a temporary cement (Temp Bond, Kerr, USA). Final metal-ceramic or zirconium single crowns were placed and cemented with a definitive powder liquid cement (Polycarboxylate, Heraeus Kulzer, Germany) after 6 months from tooth build-up. The crown rehabilitation was performed in 46 out of 68 (68%). All teeth were in occlusal contact and were not used as abutments for multiple fixed restorations.

### Extraction and implant rehabilitation (Implant group)

Extraction and implant insertion procedures were performed according to standardized clinical protocol [41–43].

For immediate placement, a 1.2 mm drill was used to prepare the alveolar socket, following the palatal bony walls as a guide. A series of calibrated drills were used under copious irrigation and at 225 rpm with sterile saline solution. Primary implant stability was obtained by anchoring the implant in the remaining apical portion of the socket at least 3 mm beyond the root apex area. Titanium implants (Prima Connex, Keystone, Burlington, MA, USA; Premium SP and Prama implants, Sweden & Martina, Padova, Italy) were placed to keep the blasted surface at cortical bone level smooth portion of the neck at soft tissue level. A 1.0 mm cover screw was then positioned and maintained for all the healing phases.

Early implant delivery was selected when the periapical infection at the moment of the extraction did not allow a correct placement. In this case, period of approximately 3 to 6 months was considered sufficient before implant placement. A 1.2 mm drill was used to mark the position, angle and depth. The drill passed through the mucosa, cortical bone and cancellous bone under copious saline irrigation. A series of twist and calibrated drills at 225 rpm was used and a site of the adequate depth and diameter was created whilst irrigating with sterile saline solution. A 1 mm cover screw was then positioned and maintained for all the healing phases.

Loading procedures were performed 3 months after implant insertion. Briefly, impressions were taken in customised resin trays with polyether materials (Permadyne and Garant, 3 M ESPE, St Paul, MN, USA). Customised abutments were positioned after 7–15 days and provisional crowns cemented in the same session with a zinc oxide eugenol cement (Temp Bond, Kerr, USA). After one-month, definitive metal-ceramic rehabilitations were luted with a polycarboxylate powder/liquid cement (Heraeus Kulzer, Hanau, Germany).

### Radiographical analysis and recall procedures

The periapical radiographs and clinical data were used to classify the final outcome in both treatments. Each patient was checked during the routine recall visit and inspected by one of two examiners for coronal/crown integrity, periapical radiographic status (Endo group) and radiographic bone level stability (Implant group). Radiographs were taken using the paralleling technique and dental intraoral films (Kodak, Rochester, NY, USA). Exposure time of each filling was standardised, and a film holder was used (Rinn Corp., Elgin, IL, USA). Radiographic evaluation was performed pre- and post-operatively, every 1–2 years, when the clinical symptoms or coronal status required a further radiographic inspection, and at the endpoint by one additional examiner blinded to the study. The following periapical radiographs were analysed by two independent examiners: before the treatment, at the moment of the treatment (root canal filling or implant insertion), at 4-year and at 8-year follow-up.

Periapical index (PAI) [44] was used to monitor the periapical lesion status and their modifications during the follow-up examinations. PAI was evaluated in single blind by two additional operators (who did not participate in the root canal treatment procedures).

### Root canal retreatment outcome

Prior to radiographic evaluation, the examiners were calibrated using pre-defined instructions and reference radiographs with various types of periapical lesions. At the endpoint, periapical tissues were classified on the basis of PAI as follows:

- *Healthy*: absence of radiographic signs of periapical lesions (PAI ≤ 2), and absence of clinical signs and symptoms including no tenderness to percussion (strict criteria). Healthy teeth determined the success rate of the study [40, 45].
- *Persistent apical periodontitis*: radiological signs of endodontic disease (PAI ≥ 3) during follow-up [40, 45].

#### Survival rate

number of healthy and endodontic lesion still functional at the end line of the study.

Complications were assessed and divided as:

- *Minor*: complications that did not affect the endodontic retreatment outcome (both success and survival) (e.g. prosthetic complications).
- *Major*: complications that affected the endodontic outcome/healing/success and required a reintervention but did not undermine tooth survival (e.g. persistent periapical lesion).
- *Critical*: complications that affected the survival rate of the tooth (e.g. fractures).

### Implant rehabilitation outcome

The crestal marginal bone and the bone-implant contact were examined to evaluate the marginal bone level (MBL). MBL was assessed at the mesial and distal implant surfaces by measuring the distance between the reference point of the implant platform to the most coronal bone-to-implant contact level using a scale divided into 0.1 mm steps and corrected according to the known height and width of each implant. Radiographic evaluation was performed in single-blind by two additional examiners. Before evaluating the radiographs, the examiners were calibrated by using well-defined instructions and reference radiographs with different marginal bone level measures.

An estimate for the *Survival rate* was calculated as the number of implants still functional at the end line of the study. A complementary *Success rate* was calculated according to traditionally accepted criteria and included lack of mobility, lack of infection or suppuration, MBL < 1.0 mm in the first year, and < 0.2 mm increase for each subsequent year [46–49].

Complications were assessed and divided as:

- *Minor*: complications that did not affect the implant outcome and survival and success (e.g. prosthetic complications);
- *Major*: complications that affected the implant radiological and aesthetic success but did not affect survival (e.g. MBL losses more than 1 mm after 1 year and 0.2 mm after subsequent years or soft tissue dehiscence with exposure of the implant neck);
- *Critical*: complications that affected the survival rate implant (e.g. peri-implantitis).

### Development of a decision-making score (DM-score)

A numerical score was retrospectively constructed to define and create a score for future clinical applications. Various scoring values were assigned to the structural, periodontal and endodontic parameters previously recorded (Table 1). The Decision-Making Score was developed to define strict clinical criteria supporting the operator during the decision-making step.

**Table 1:** Decision making score used to establish the endo-group and Implant-group

	Value	Coefficient	Output
*Structural Parameters*			
Residual coronal structure			
3 or 4 residual walls	+ 1	+ 4	+ 4
1 or 2 residual walls	−1	+ 4	−4
No residual walls	−2	+ 4	−8
Full reconstruction	−2	+ 4	−8
Deep carious lesions			
No	+ 1	+ 2	+ 2
Yes	−1	+ 2	−2
*Periodontal Parameters*			
CEJ–MBL distance, mm			
≤ 3	+ 1	+ 3	+ 3
> 3 to < 6	−1	+ 3	−3
≥ 6	−2	+ 3	−6
*Endodontic Parameters*			
Initial PAI			
1 or 2	−1	+ 2	−2
3 or 4 5	+ 1 −1	+ 2 + 2	+ 2 −2
Preoperative post			
No	+ 1	+ 2	+ 2
Yes	−1	+ 2	−2

Notes: Negative values lead to higher indications for tooth extractions; positive values lead to higher indications for secondary retreatment. Coefficients were constructed in order to establish a “priority/gravity” for each parameter. A higher coefficient corresponds to a lower indication for secondary endodontic treatment.

Based on data from the literature, coefficients of importance/severity were applied to the parameters that may critically affect the tooth prognosis/survival, guide the operator to the clinical decision and influencing the tooth prognosis towards extractions.

These include reduced residual coronal structure (× 4) [22], periodontal bone loss (× 3) [24], presence of a deep carious lesion (× 2), and presence of intraradicular post (× 2) [7, 22, 50] and presence of a periapical lesion (× 2). Following literature guidelines and prior decision-making studies [28, 50, 51], higher coefficients were assigned to factors posing a greater risk of failure, undermining tooth survival (coefficients 4 and 3). Lower coefficients were assigned to factors inducing a minor risk to tooth survival. The presence of a previous post was not scored with a high priority due to the standardized endodontic protocol and operators’ endodontic expertise.

Each parameter was assigned specific values (+ 1; -1; -2) reflecting the severity of tooth compromise or endodontic disease. Positive values indicate a higher likelihood of successful resolution with root canal retreatment, while negative values indicate a lower likelihood of improvement with retreatment. The total output (Value × Coefficient) was finally calculated. Positive values (closer to 0) indicated better prognosis and favoured secondary root canal retreatment (Endo group) while negative values indicated a worse prognosis and favoured extraction (Implant group).

As a result, implant-rehabilitation mean score was − 5.6 ± 5.4 while root-canal-retreatment mean score was 0.4 ± 5.3, with a statistically significant difference of − 5.9 (95% CI − 7.8 to − 4.0, P-value < 0.001).

### Statistical analysis

Numerical variables were summarised as mean ± standard deviation; categorical variables were summarised as frequencies and percentages. Crude differences in baseline characteristics between the two treatment groups were assessed by means of simple logistic regression analysis with clustered standard errors to allow for intragroup correlation within teeth belonging to the same patient. Results were expressed for each variable as odds ratios (ORs) of implant rehabilitation to root canal retreatment with 95% confidence intervals (CIs). A similar approach was used to compare mean decision-making scores, but a linear model was used in place of a logistic model.

Survival and treatment success for the two study groups were estimated using the Kaplan–Meier method using the date of surgery as the time origin and treating losses to follow-up as right-censored data. The association of treatment group with time to event was assessed using Cox proportional hazard regression analysis with clustered standard errors to allow for intragroup correlation within teeth belonging to the same patient. Results were expressed as hazard ratios (HRs) of experiencing the study event among implants as compared to root canal retreatments with 95% CIs. The proportional-hazards assumption was confirmed after checking for nonzero slope of scaled Schoenfeld residuals on time.

In a sensitivity analysis, Cox regression was rerun using weights based on propensity-for-treatment scores in order to fully balance baseline demographic and anatomical characteristics in the two study groups. Multiple Additive Regression Trees (MART) gradient boosting was used to estimate the propensity to be assigned to one treatment or the other with a set of explanatory variables including sex, age, tooth location, and tooth type. As a rule of thumb, the following settings were adopted for regularisation: maximum tree depth of 5 interactions; maximum of 20,000 iterations; 50% bagging; 0.01 shrinkage factor. Each observation was weighted by the reciprocal of the probability of receiving the treatment that was actually received, which is known as inverse probability treatment weighting (IPTW). Weights were truncated at the 99th percentile. Mean differences in PAI (for Endo group) and MBL (for Implant group) were estimated with linear regression analysis with clustered standard errors.

All analyses were carried out using Stata software, version 17 (StataCorp, College Station, TX, USA). The significance level was set at 5%, and all tests were two-sided.

### Power analysis

The Endo-group 8-year survival rate was estimated at 80%. An anticipated improvement in survival for the Implant group, corresponding to a hazard ratio of 0.10 (approximately 98% 8-year survival rate), was to be detected with 80% power using a two-sided log-rank test at a 0.05 significance level, assuming 1:1 allocation. A total of 12 events (failures) were required to achieve 80% power for detecting a hazard ratio of 0.10. Based on this, the estimated total number of units needed to observe 12 events was 106, with a minimum of 53 units per group.

## Results

A total of 96 patients (49 males and 47 females, mean age 53.1 ± 11.6 years) were considered eligible in the study. After the pre-operative clinical evaluation, retreatments were performed in 68 teeth (54.8%) as Endo group. Extractions followed by implant placements were effectuated in 56 teeth (45.2%) and included in the Implant group (Table 2).

**Table 2:** Baseline characteristics of the premolars included in the study (*n* = 124)

	*n* (%)
*Patient Characteristics*	
Sex	
Male	63 (50.8%)
Female	61 (49.2%)
Age group, y	
<60	88 (71.0%)
≥60	36 (29.0%)
*Anatomical Characteristics*	
Tooth location	
Maxilla	85 (68.5%)
Mandible	39 (31.5%)
Tooth type	
First premolar	47 (37.9%)
Second premolar	77 (62.1%)
*Structural Characteristics*	
Coronal integrity	
Prosthetic crown	40 (32.3%)
No residual walls	15 (12.1%)
1 residual wall	19 (15.3%)
2 residual walls	17 (13.7%)
3 or 4 residual walls	33 (26.6%)
Deep carious lesion	
No	70 (56.5%)
Yes	54 (43.5%)
*Periodontal Characteristics*	
CEJ–MBL distance, mm	
≤3	68 (54.8%)
>3 to < 6	46 (37.1%)
≥6	10 (8.1%)
*Endodontic Characteristics*	
PAI at baseline	
1 or 2	40 (32.3%)
3 to 4 5	82 (66.2%) 2 (1.7%)
Preoperative intra-canal post	
No	74 (59.7%)
Yes	50 (40.3%)

### Baseline characteristics and likelihood of endo vs. implant

Baseline characteristics of pre-operative parameters of teeth are summarised in Tables 3a and 3b. No significant differences in demographic and anatomical characteristics were found between the two groups (Table 3a).

**Table 3:** **a.** patient and anatomical characteristics of premolars after clinical decision making and dichotomized in endo-group or Implant-group

	Endo group (*n* = 68)	Implant group (*n* = 56)	Odds Ratio (95% Confidence Interval)	*P*-value
*Patient Characteristics*				
Sex				
Male	31 (45.6%)	32 (57.1%)	Ref.	
Female	37 (54.4%)	24 (42.9%)	0.63 (0.29, 1.38)	0.246
Age group, y				
< 60	52 (76.5%)	36 (64.3%)	Ref.	
≥ 60	16 (23.5%)	20 (35.7%)	1.81 (0.79, 4.13)	0.161
*Anatomical Characteristics*				
Tooth location				
Maxilla	49 (72.1%)	36 (64.3%)	Ref.	
Mandible	19 (27.9%)	20 (35.7%)	1.43 (0.63, 3.28)	0.395
Tooth type				
First premolar	27 (39.7%)	20 (35.7%)	Ref.	
Second premolar	41 (60.3%)	36 (64.3%)	1.19 (0.61, 2.30)	0.615

*P-value ≤ 0.05

**Table 3:** **b.** structural, periodontal, and endodontic characteristics in endo-group vs. Implant-group)

	Endo group (*n* = 68)	Implant group (*n* = 56)	Odds Ratio (95% Confidence Interval)	*P*-value
*Structural Characteristics*				
Coronal integrity				
Prosthetic crown	15 (22.1%)	25 (44.6%)	Ref.	
No walls	3 (4.4%)	12 (21.4%)	2.40 (0.57, 10.14)	0.234
1 residual wall	11 (16.2%)	8 (14.3%)	0.44 (0.14, 1.36)	0.153
2 residual walls	15 (22.1%)	2 (3.6%)	0.08 (0.02, 0.41)	0.002*
3 or 4 residual walls	24 (35.3%)	9 (16.1%)	0.23 (0.08, 0.65)	0.006*
Deep carious lesion				
No	30 (44.1%)	40 (71.4%)	Ref.	
Yes	38 (55.9%)	16 (28.6%)	0.32 (0.14, 0.69)	0.004*
*Periodontal Characteristics*				
CEJ–MBL distance, mm				
≤ 3	42 (61.8%)	26 (46.4%)	Ref.	
> 3 to < 6	25 (36.8%)	21 (37.5%)	1.36 (0.59, 3.10)	0.470
≥ 6	1 (1.5%)	9 (16.1%)	14.5 (1.60, 132.2)	0.017*
*Endodontic Characteristics*				
PAI at baseline				
1 or 2	8 (11.8%)	32 (57.1%)	Ref.	
3 to 5	60 (88.2%)	24 (42.9%)	0.10 (0.04, 0.28)	< 0.001*
Preoperative intra-canal post				
No	46 (67.6%)	28 (50.0%)	Ref.	
Yes	22 (32.4%)	28 (50.0%)	2.09 (0.92, 4.76)	0.079

*P-value ≤ 0.05

Conversely, significant differences were observed in relation to baseline parameters such as residual coronal structure, deep carious lesions, CEJ–MBL distance and PAI. In particular, ≥ 2 residual dentine walls, deep carious lesions and PAI values ≥ 3 were associated with increased likelihood of root canal retreatment, while preoperative CEJ–MBL distance ≥ 6 mm was associated with increased likelihood of replacement with dental implant (Table 3b).

### Outcome measures

#### Survival rate in Endo and Implant groups

As shown in Table 4, the 8-year survival rates of Endo and Implant groups were 85.1% (95% CI 74.0–91.7%) and 98.2% (95% CI 88.0–99.8%), respectively, with a HR resulting from Cox regression equal to 0.12 (95% CI 0.01 to 0.99, *P*-value = 0.049) favouring implant treatment. Specifically, critical complications in the Endo group were due to fractures in eight cases (12%), periodontal lesions in one and recurrent endodontic lesion in one case. In the Implant group, a critical complication for periimplantitis was observed after six months.

**Table 4:** Eight-year survival and success rate (%) for Endo vs. Implant-group

	Endo group		Implant group	
	Est.	95% CI	Est.	95% CI
Survival	85.1	74.0, 91.7	98.2	88.0, 99.8
Success	80.5	68.8, 88.2	93.9	82.3, 98.0

Notes: Success for root canal retreatments is no extraction or reintervention, while success for implants is no extraction, biological complication, mucositis or bone loss > 2 mm

*CI*, confidence interval

### Success rate

**Fig. 2:**
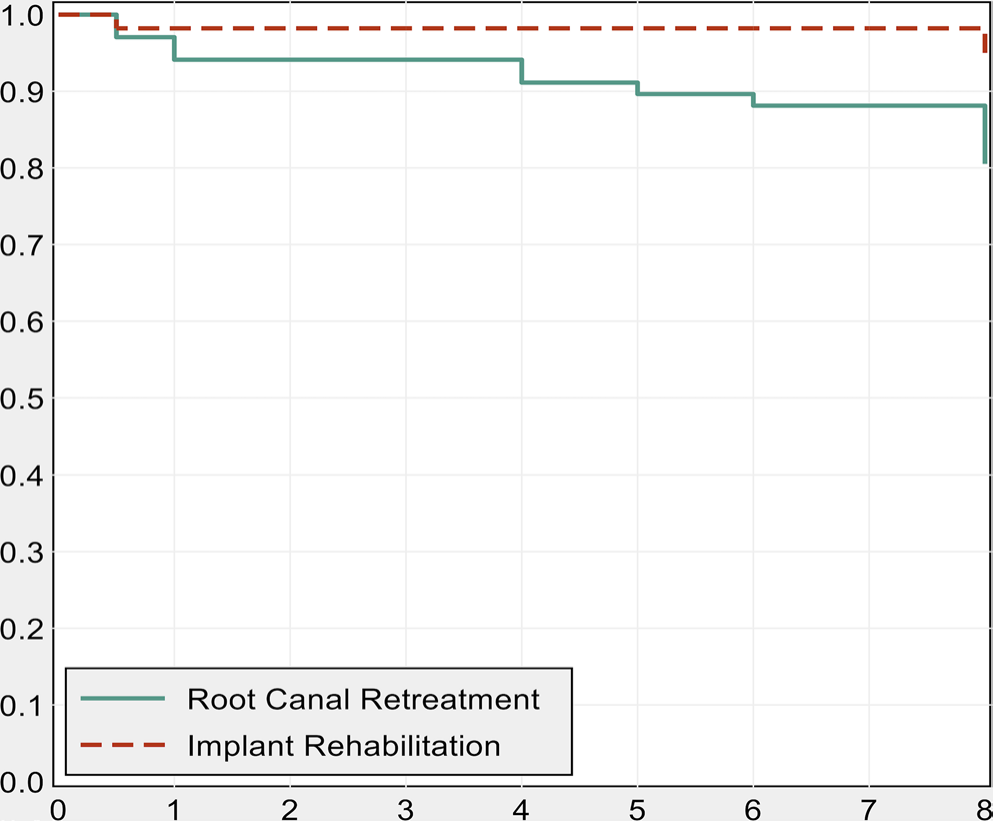
Kaplan–Meier survivor estimates of time to critical or major complications after surgical treatment in retreated vs. extracted and implanted premolars.

As shown in Table 4; Fig. 2, the eight-year success rate of Endo and Implant-group was 80.5% (95% CI 68.8–88.2%) and 93.9% (95% CI 82.3–98.0%), respectively, with a HR resulting from Cox regression equal to 0.28 (95% CI 0.08 to 1.04, *P*-value = 0.057) in favour of Implant group. Specifically, in addition to the ten extractions listed earlier, one premolar underwent a surgical apicoectomy and two were additionally retreated due to presence of persistent periapical lesions (PAI 4).

### Complications

**Fig. 3:**
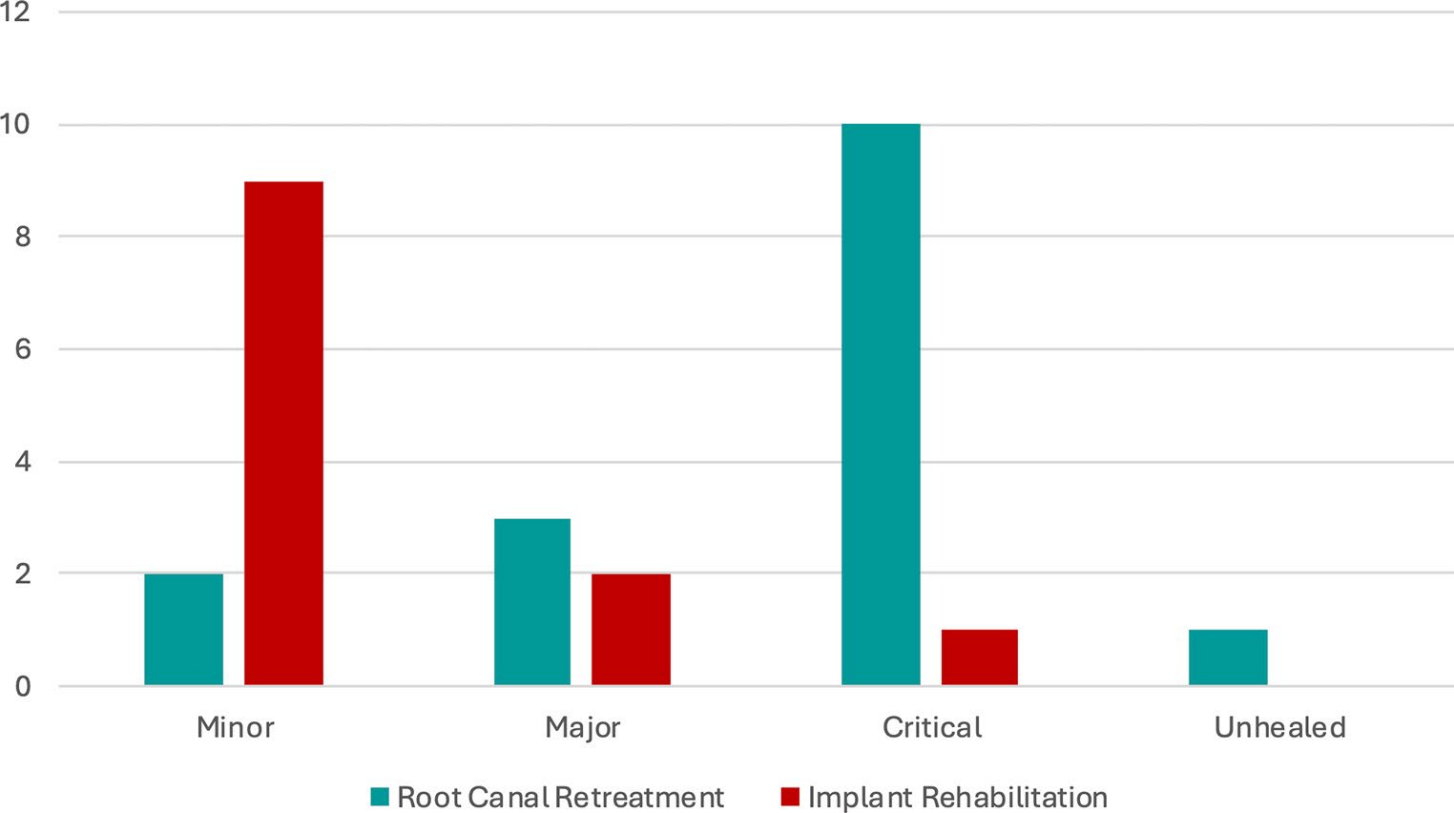
Graph reporting complications/events occurred during the eight-year follow-up in Implant rehabilitation and Root canal Retreatment group. Minor complications include prosthetic complications; major complications include periapical lesion re-exacerbations requiring a reintervention; critical complications include teeth extracted for root fracture, periodontal loss, and endodontic reasons (fistula and persistent symptomatology). Unhealed teeth are teeth with a stable asymptomatic lesion.

In the Endo-group, critical and major complications resulted higher in comparison to the Implant group (Fig. 3). Two minor prosthetic complications were observed in the Endo-group (2 out of 68).

Table 5 reports the number of healed teeth, extractions, and unhealed teeth in the Endo-group according to structural, periodontal, and endodontic parameters. Critical complications (followed by extractions) occurred more frequently when residual crown structure was compromised.

**Table 5:** Number and percentages of healthy, unhealed, and extracted teeth in the Endo group according to structural, periodontal, and endodontic parameters after 8 years of follow-up

	Healed/healthy (n* = *54)	Unhealed/Reintervention (*n* = 4)	Lost during follow-up (*n* = 10)	Unhealed/reintervention vs. Healed/healthy		Lost during follow-up vs. Healed/healthy	
				OR (95% CI)	*P*-value	OR (95% CI)	*P*-value
*Structural Characteristics*							
Coronal integrity							
Prosthetic crown	11 (73.3%)	1 (6.7%)	3 (20.0%)	Ref.			
No walls	2 (66.7%)	1 (33.3%)	0 (0.0%)	4.69 (0.05, > 100)	0.371	1.76 (0.02, 46.79)	1.000
1 residual wall	7 (63.6%)	0 (0.0%)	4 (36.4%)	1.54 (0.02, > 100)	1.000	2.03 (0.26, 18.44)	0.656
2 residual walls	13 (86.7%)	1 (6.7%)	1 (6.7%)	0.85 (0.01, 72.39)	1.000	0.29 (0.00, 4.29)	0.596
3 or 4 residual walls	21 (87.5%)	1 (4.2%)	2 (8.3%)	0.53 (0.01, 44.82)	1.000	0.36 (0.03, 3.64)	0.346
Deep carious lesion							
No	21 (70.0%)	2 (6.7%)	7 (23.3%)	Ref.			
Yes	33 (86.8%)	2 (5.3%)	3 (7.9%)	0.64 (0.04, 9.48)	1.000	0.28 (0.04, 1.39)	0.090
*Periodontal Characteristics*							
CEJ–MBL distance, mm							
≤ 3	32 (76.2%)	3 (7.1%)	7 (16.7%)	Ref.			
> 3 to < 6	21 (84.0%)	1 (4.0%)	3 (12.0%)	0.51 (0.01, 6.90)	0.652	0.66 (0.10, 3.30)	0.729
≥ 6	1 (100.0%)	0 (0.0%)	0 (0.0%)	9.38 (0.10, > 100)	0.207	4.34 (0.05, > 100)	0.356
*Endodontic Characteristics*							
PAI at baseline							
1 or 2	8 (100.0%)	0 (0.0%)	0 (0.0%)	Ref.			
3 to 4	45 (76.3%)	4 (6.8%)	10 (16.9%)	0.72 (0.06, 39.35)	1.000	1.76 (0.19, 86.67)	0.692
5	1 (100.0%)	0 (0.0%)	0 (0.0%)	6.00 (0.05, > 100)	0.346	6.00 (0.05, > 100)	0.346
Preoperative intra-canal post							
No	38 (82.4%)	3 (6.7%)	4 (8.9%)	Ref.			
Yes	16 (69.6%)	1 (4.3%)	6 (26.1%)	0.79 (0.01, 10.79)	1.000	3.48 (0.72, 19.21)	0.080

Notes: Due to small sample sizes, statistical inference was conducted using exact logistic regression analysis, which produces conditional maximum likelihood estimates

In the Implant group, two major complications (bone losses > 2 mm in the 4–8 years follow-up) were observed. During the follow-up, eight implant rehabilitations showed minor complications that led to a prosthetic reintervention, namely five recurrent abutment loosening, two coronal chipping and one recurrent crown decementation (8 out of 56 [14.3%]).

Propensity-score sensitivity analysis confirmed a borderline-significantly lower risk of incurring in biological complications or extractions (i.e., better healing) in the Implant group as compared to the Endo-group (HR 0.31, 95% CI 0.08 to 1.17, *P*-value = 0.084). Similarly, difference in tooth/implant survival failed to achieve statistical significance (HR 0.13, 95% CI 0.02 to 1.06, *P*-value = 0.057).

### PAI evaluation in the Endo group

Cross tabulation of initial versus final PAI in the Endo-group was reported in Table 6. Among the 68 root canal retreatments that did not incur into critical complications, mean baseline PAI was 3.2 (95% CI 3.0 to 3.4) while mean eight-year PAI was 1.1 (95% CI 1.03 to 1.24), with a statistically significant mean reduction of 2.1 points (95% CI 1.8 to 2.3, *P*-value < 0.001).

**Table 6:** Cross-table of final PAI against initial PAI of teeth which underwent root canal retreatment. Extractions were added as an ultimate sixth stage

	Final PAI 8 years (*n*)					
	Healthy		Endodontic lesion			Extractions
Initial PAI	1	2	3	4	5	
1 (*n* = 1)	1	-	-	-	-	-
2 (*n* = 7)	6	1	-	-	-	-
3 (*n* = 37)	26	4	-	-	-	7
4 (*n* = 22)	17	1	1	-	-	3
5 (*n* = 1)	1	-	-	-	-	-

### MBL evaluation in the implant group

**Fig. 4:**
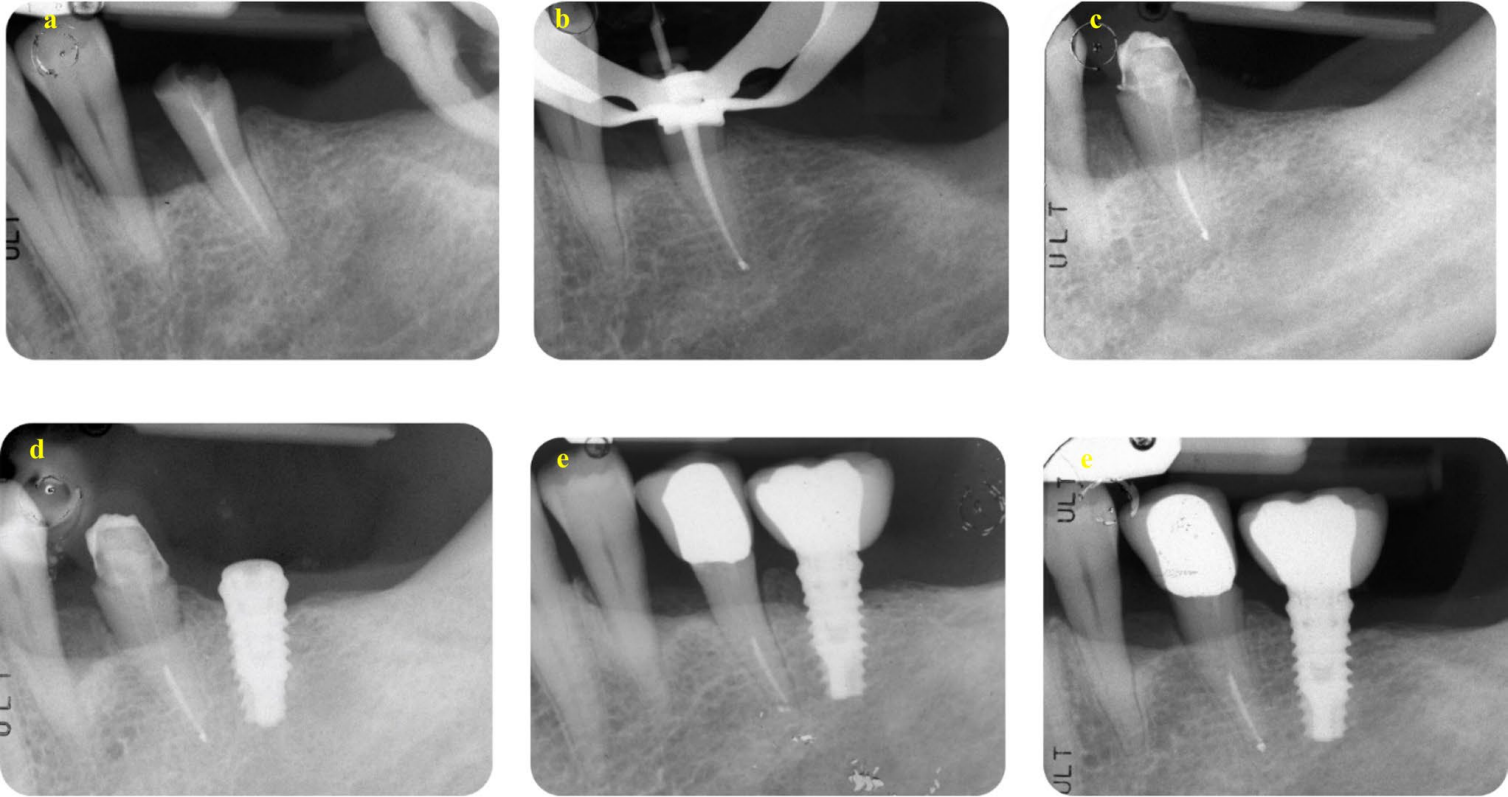
A representative case on the Endo group. (**a**) The DM-Score was + 1 and suggested a root canal retreatment. Score was calculated as follows: no residual walls (-8), no deep carious lesion (+ 2), CEJ MBL distance was less than 3 mm (+ 3), initial PAI was 3 (+ 2) and no preoperative post was present (+ 2). (**b**) Root canal obturation was made using a carrier-based technique associated to an epoxy-resin based sealer. (**c**) post endodontic reconstruction was performed after 14 days from filling. (**d**) A carbon post was inserted and tooth was prepared to receive a provisional crown that was maintained up to 6 months. (**e**) After 6 months, a definitive crown was cemented. (**f**) After 8 years, periapical tissues are stable and healthy, with no modifications

**Fig. 5:**
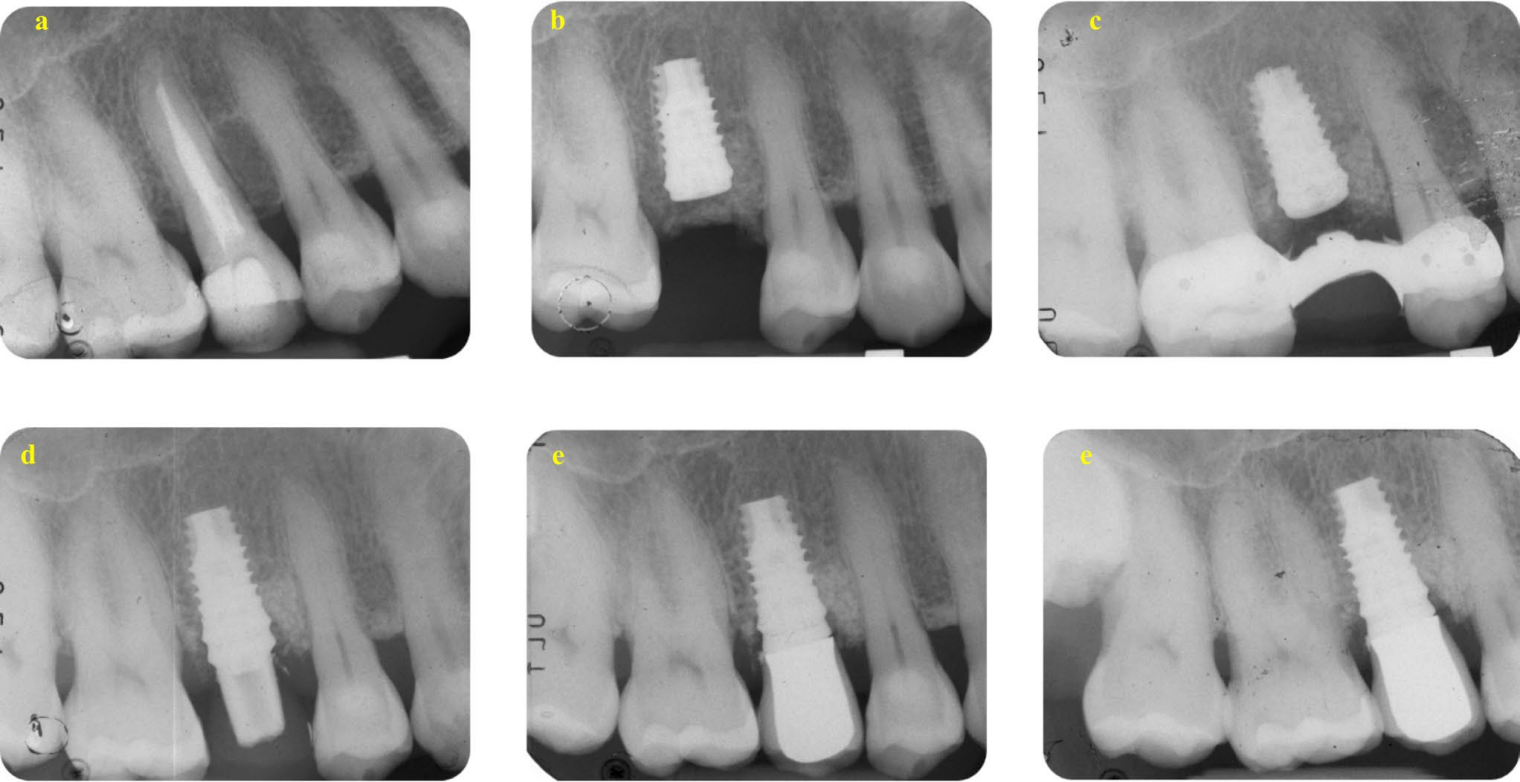
A representative case of the Implant group. (**a**) The DM-Score was − 5 and suggested an extraction and implant placement. Score was calculated as follows: 2 residual (non-infiltrated) walls (-4), deep carious lesion (-2), CEJ MBL distance was less than 3 mm (+ 3), initial PAI was 2 (-2) and no presence of a preoperative post (+ 2). (**b**) Extraction was performed and early implant insertion was scheduled due to the presence of a periapical infection. (**c**) A cemented-retained Maryland bridge had been positioned as “interim prosthesis” for all the healing period. (**d**) After 3 months a customised abutment and a provisional crown was positioned. (**e**) After 28 days, a definitive metal ceramic crown was cemented. (**f**) Periapical radiographs after 8 years showed MBL stability

The distribution of MBL classes is presented in Table 7. Among the 56 implants, mean MBL at four years was 0.95 mm (95% CI 0.82 to 1.09) and 1.25 mm at eight years (95% CI 1.09 to 1.40), respectively, with a statistically significant MBL increase of 0.29 mm (95% CI 0.19 to 0.40, *P*-value < 0.001). Representative cases of teeth included in the study with an 8-year follow-up are shown in Figs. 4 and 5.

**Table 7:** Percentage distribution of marginal bone level (MBL) values (mm) of implants at four and eight years of follow-up

Grouped MBL at 4 years	Total (*n* = 56)	Grouped MBL at 8 years			
		0 to 1 mm	> 1 mm to 2 mm	> 2 mm to 3 mm	Lost to FUP*
		(*n* = 28)	(*n* = 24)	(*n* = 2)	(*n* = 2)
0 to 1 mm	43 (77%)	28 (100%)	13 (54%)	0 (0%)	2 (100%)
> 1 mm to 2 mm	12 (21%)	0 (0%)	11 (46%)	1 (50%)	0 (0%)
> 2 mm to 3 mm	1 (2%)	0 (0%)	0 (0%)	1 (50%)	0 (0%)

*Patients prematurely deceased within 3 and 4 years of surgery

## Discussion

This non-randomized historical prospective clinical study evaluated the outcome of two different approaches in the rehabilitation of compromised endodontically-treated premolars. All participants were obtained among a pool of patients sent for decision making at the University Endodontic Department to obtain a definitive selection of the therapy. The strict inclusion criteria adopted in this study minimized clinical variabilities affecting tooth anatomies and root canal morphologies. The study also evaluated the impact of pre-operative parameters on clinical outcome. The type and frequency of complications, as well as the progression and number of failure events in both clinical treatments were analysed over a minimum 8-year period.

The study demonstrated statistically higher survival rates of Implant-group (98%) with respect to the Endo-group (85%). Clinical investigations which dedicated attention on implants survival rate at 8–10 years [52–55] are aligned with the results collected by this comparative study. Investigations on retreatment strategies demonstrated percentage of clinical and radiographical success not far from those reported by the present study [9, 24, 35, 56]. The success rate of retreatments at 8–10 years was reported ranging between 75 and 85%, a lower percentage with respect the replacement strategy.

The filling technique and type of sealer used, namely carrier-based approach associated to epoxy resin-based sealer, was chosen as considered the gold standard at the moment of treatment performance. Additionally, it was selected due to its rapid learning curve and ease of adoption in postgraduate clinical practice [9]. Previous studies evidenced a good marginal seal, deep penetration of the sealer in dentinal tubules [57] and higher flow of epoxy resin-based sealers compared to other materials [58]. Recent systematic reviews and meta-analyses also confirmed the stability and the validity of epoxy resin based obturations compared to other more recently introduced sealers [59, 60].

The primary causes of clinical failures reported in these investigations were predominantly non-endodontic, such as root fractures and losses attributed to periodontal conditions [10, 13, 19, 24]. In several studies the percentage of extractions was documented but specific data on failure causes were not provided [35, 56]. A previous systematic review and meta-analysis also reported a percentage of clinical success of 76% at 4 years [6], a critical time for endodontic treatments according to international guidelines [61]. Considering all these factors, we decided to assess the treatment outcomes over a longer timeframe, specifically with an 8-year follow-up. The main clinical parameter adopted in our study for Endo-group was the PAI score, as it reliably indicates the status of bone periapical lesions [62]. It should be specified that a complete success (healing) of the secondary root canal treatment (according to strict criteria) is more difficult to obtain when compared to implant rehabilitations, as it implies the healing of a previous periapical lesion (PAI > 2), absence of pain, symptomatology and absence of any endodontic complications during follow-up. In addition, the longevity of coronal seal, integrity of direct and indirect restorations, factors not related to the secondary endodontic treatment, could also critically affect the final outcome [6, 7]. In the Endo group, a higher presence of functional teeth could be expected in 10–20 long term follow-ups, teeth that shows a persistent periapical radiolucency (teeth with apical scar formation) but remain clinically asymptomatic [8, 24, 45]. These teeth are usually considered as failed endodontic cases (according to strict criteria) but successful in accordance to “loose criteria”.

In the implant group it is less frequent to observe such situation. Indeed. cases with increased MBL in the first months from implant insertion usually end up with a lower long-term prognosis [48, 49]. These data confirm that implant success is highly-related to the bone stability of the first months osseointegration events (type of implant placement and loading). Marginal bone loss (MBL), used as an indicator of osseointegration [46–48], provided strong evidence of long-term peri-implant bone stability. The findings from this study support the conclusion that most of implant procedures were free from significant complications such as bone defects, gingivitis, and peri-implantitis. Our study showed that prosthetic protocols and loading procedures were not responsible for extensive bone loss, which could have affected the long-term follow-up period. Appearance of peri-implant bone defects occurred in a small number of patients, in line with traditional success criteria [63, 64]. It is important to note that implants were placed in extraction sockets of endodontically-treated premolars which presented a previous periapical lesion (abscess or chronic periapical disease with recurrent infections). This condition is frequent in an Endodontic Department and represents a complex clinical scenario. The bone quality in extraction sites affected by periapical lesion (i.e. acute periapical lesion) has been evaluated by other investigations [65–70]. The risk for periimplantitis and early failures associated with bacteria permanence and/or bone defect healing is critical [65–70]. Implants placed in post-endodontic sites usually presented higher risk for bone loss and periimplantitis in the early time [65, 67]. In our study, one case of peri-implantitis was observed after six months. The careful evaluation of the socket, standardization of surgical procedures, and inclusion in a recall program may have positively influenced the overall outcomes. Notably, the different implant types used in this investigation did not impact marginal bone loss (MBL), despite variations in implant neck morphology and surface design.

In the Endo-group, root fracture was the most common complication, mostly occurring after four years, as shown in the Kaplan-Meier analysis. The presence of one or less residual walls and the presence of a previous prosthetic crown were identified as the most critical factors associated with root fractures. The significance of preoperative structural integrity of crown has been highlighted in another clinical study [22]. Root fracture was more frequently observed when less than 30% of the original tooth structure was present before the root canal retreatment [22]. The clinicians must keep in account this biomechanical limit [71, 72]. Another explanation may be in relationship with the long-term activity of root canal irrigant solutions inducing collagen degradation [73, 74]. The extensive reduction of the canal walls by shaping protocols after treatment and retreatment procedures [75] and the intrinsic fragility of premolar roots are other possible explanations of long-term mechanical failures. Hence, the study makes evident that root fractures more than endodontic failures (i.e. not healed periapical lesion) are the main reasons for clinical complications occurred during the 8-year observation period [76]. The presence of apical re-exacerbation during the follow-up, followed with clinical symptomatology was a very low event (5.8%) requiring an endodontic reintervention. An additional retreatment was performed in cases where the endodontic space was still accessible without compromising the overall integrity of the tooth, such in cases of new metal-ceramic or zirconia rehabilitations. In this case (one tooth), surgical apicoectomy allowed to remove the apically infected area and to resolve the symptomatology. Surgical apicoectomy was therefore performed only when the orthograde retreatment is not feasible [77], offering a more favorable early success, but a less favorable long-term outcome [25]. It is evident that the presence of a pre-operative PAI indicative for periapical lesion did not represent a critical factor that guided the pre-operative decision. Previous studies well described the different approach deserved by endodontist or other clinician to the management of endodontically complex cases [3, 28].

Prosthetic complications (recurrent crown decementation or coronal chipping) were included as minor complications, being conditions not responsible for rehabilitation longevity. Issues were primarily observed in the Implant-group, as 15% of crowns were affected. In contrast, limited prosthetic complications (3%) were noted in the Endo-group. It is well-known that implant rehabilitations may present prosthetic complications in the long-term i.e. abutments screw loosening or coronal chipping [78].

As a secondary aim, the study retrospectively analysed the pre-operative parameters that influenced the clinical decision to select one of the treatments (Endo or Implant groups). In this way it was possible to re-evaluate the correctness of choice that guided the preoperative decision.

Interestingly, the presence of an intraradicular post was not a significant parameter that influenced the clinical decision making (*p* > 0.050), despite the fact that post removal could be a significant aspect for the treatment sequence. The removal of a post may increase the risk of failure in the long term in non-skilled operators [79]. However, the adopted clinical protocol and operator expertise may have influenced final outcome [80, 81]. Previous studies confirmed this trend, showing that endodontic specialists were more skilled and trained than general dentists to perform a root canal retreatment in presence of an endodontic post [3] and that different decision-making could be expected when in presence of endodontically affected tooth with crown deficit/alterations [3, 30, 82].

Root canal secondary treatment for premolars may have higher chances of failure compared to anterior groups, possibly due to their reduced coronal structure and higher variability of the root canal morphology [82, 83]. Premolars present the highest incidence of vertical root fractures [84, 85], have the smallest mesiodistal root diameter [84, 86] and high palatal and buccal stress concentration areas during function [86, 87]. The greater risk of failure of premolars has been also recently highlighted in a retrospective investigation on over one million endodontic treatments, reporting lower survival rates of premolars (approx. 90%) when compared to anterior teeth (approx. 95%) after 11 years of follow-up [88]. The choice to have restricted the analysis to these teeth must be considered. Their intrinsic “fragility” could have influenced the results. The high 8-year survival rate of both Endo and Implant group, comparable to the values reported in literature [9, 24, 40, 54] confirms the reliability of the decision making performed by the operators and could support clinical applicability of the conceived DM-score in identifying the most appropriate treatment.

As final consideration, this study focused exclusively on teeth affected by periapical lesions and previously treated with endodontic procedures—a common occurrence in clinical practice. The pre-operative condition of these teeth was so compromised that both treatment options—secondary root canal therapy or extraction with implant placement—were considered ethically-acceptable. Both options are complex procedures requiring a highly skilled and well-coordinated team with extensive knowledge in prosthetic rehabilitation. Collecting and evaluating clinical parameters to inform the pre-operative decision-making process is a complex and detailed task. The experience and protocol expertise of the operator play a critical role, necessitating a high level of skills.

The study has some limitations. The retrospective analysis of decision-making may involve recall bias, and the 8-year follow-up might not capture very long-term outcomes (10 to 20 years), especially for implants. Finally, the operator expertise and standardized protocols may not reflect general practice in non-university set ups. Further randomized trials with longer follow-ups and diverse populations are needed to validate these findings.

## Conclusions

This 8-year clinical study demonstrated that:

1. Endo-group showed greater critical and major complication that lead to lower percentage of teeth in clinical function (85.1%) with a success rate of 80.5%. These percentages were in line with literature data.
2. The main reason for Endo-group failures was based on the high number of root fractures observed after 4–8 years. It is not a failure of endodontic therapy per se but is a consequence of reduced crown structure and retreatment irrigation protocols.
3. Implant-group displayed less critical complications during the follow-up and higher survival rate (98%).
4. The strategy to replace teeth with implants led lower critical or major complications but higher prosthetic complications.

The management of endodontically treated teeth and still affected by periapical lesion and by coronal alterations require great attention. A multidisciplinary approach is therefore required to treat such complex cases [89].

## Data Availability

Data cannot be shared openly but are available on request from authors.
